# Efficacy of Continuous Infusions of Furosemide-Albumin Compared With Bumetanide in Critically Ill Patients: A Retrospective Cohort Study

**DOI:** 10.1177/10600280261425654

**Published:** 2026-03-30

**Authors:** Sara Hussein, Hala Halawi, Mahmoud Sabawi

**Affiliations:** 1 Division of Pharmacy, The University of Texas at MD Anderson Cancer Center, Houston, TX, USA; 2 Department of Pharmacy, Houston Methodist Hospital, Houston, TX, USA; 3 Department of Pharmacy, Houston Methodist Willowbrook Hospital, Houston, TX, USA

**Keywords:** furosemide-albumin, bumetanide, fluid overload, critical care, diuretics, diuresis, natriuresis

## Abstract

**Background:**

Continuous loop diuretics are commonly used for management of fluid overload in critically ill patients; however, diuretic resistance remains a challenge. Comparative data on the efficacy of continuous infusions of bumetanide versus furosemide-albumin in patients requiring diuresis are limited.

**Objective:**

This study aimed to assess the effects of continuous bumetanide versus continuous albumin-furosemide infusions on urine output in critically ill patients.

**Methods:**

This was a retrospective, single health-system cohort study conducted in critically ill adult patients who received continuous bumetanide or continuous albumin-furosemide infusions for at least 24 hours. The primary endpoint was cumulative urine output at 24 hours after diuretic initiation. Secondary endpoints included total diuretic dose, net fluid balance, total albumin administered, changes in serum creatinine, and serum albumin at 24 hours following diuretic initiation, intensive care unit (ICU) length of stay, and in-hospital mortality.

**Results:**

Among the 434 patients included, 173 patients received a continuous bumetanide infusion, and 261 patients received a continuous furosemide-albumin infusion. There was no significant difference in urine output at 24 hours between the bumetanide and furosemide-albumin group (3578 mL [1818-4738] vs 3617 mL [2035-4703]; *P* = 0.304). However, patients in the bumetanide group received a significantly higher total diuretic dose in intravenous furosemide equivalents (481 mg [295-544] vs 177 mg [109-236]; *P* < 0.001) with no difference in change in serum creatinine (0.08 mg/dL [–0.19 to 0.34] vs 0.10 mg/dL [–0.07 to 0.21]; *P* = 0.597). Total albumin dose was higher in the furosemide-albumin group (10.9 g [0-12.5] vs 43.7 g [27.5-58.9]; *P* < 0.001).

**Conclusion and Relevance:**

This is the first study to evaluate continuous infusions of bumetanide versus furosemide-albumin in critically ill patients requiring diuresis, observing a comparable urine output between diuretics. Findings indicate that bumetanide may be a reasonable alternative in fluid management.

## Background

Critically ill patients commonly develop fluid overload due to disease conditions, such as heart failure, cirrhosis, renal insufficiency, and fluid administration as part of resuscitation strategies.^1^ Fluid accumulation may lead to impaired oxygenation and reduced cardiac output, and has been associated with worse outcomes, including an increased risk of mortality by 19% for every liter of excess fluid accumulated in critically ill patients.^2,3^ The cornerstone for managing fluid overload is fluid restriction and diuresis, particularly using loop diuretics.^2^ Loop diuretics exhibit their pharmacological effect by inhibiting sodium, potassium, and chloride reabsorption in the loop of Henle to promote fluid and electrolyte excretion. In turn, their clinical effect relieves hemodynamic stress and mitigates complications associated with volume overload.^4^

An ongoing challenge in the management of fluid overload with loop diuretics is diuretic resistance, where increasing doses fail to achieve the desired therapeutic effect.^4,5^ One approach to overcoming this is the use of continuous diuretic infusions in place of intermittent boluses to maintain adequate drug levels.^3,5^ A recent study demonstrated a higher urine output at 24 hours in intensive care unit (ICU) patients receiving continuous furosemide infusion compared with intermittent loop diuretic boluses (β = 732, 95% confidence interval [CI] 669-795, *P* < 0.001).^6^ Diuretic resistance may also stem from diminished renal perfusion, enhanced sodium reabsorption, and consequently decreased drug delivery to the site of action, particularly in patients with hypoalbuminemia.^4,5,7^

Since all loop diuretics are highly protein-bound, low serum albumin levels, defined as serum albumin less than 3.5 g/dL, may reduce the amount of protein-bound diuretic available for tubular secretion, thereby diminishing diuretic response.^4,7^ Specifically, furosemide is 95% albumin bound. In cases of hypoalbuminemia, the volume of distribution increases leading to a reduced amount of albumin-bound furosemide available for delivery to the proximal tubule to interact with an anion transporter and reach the loop of Henle to produce a clinical effect.^4,8^ It has been hypothesized that co-administering furosemide with albumin may enhance both diuresis and natriuresis.^4,9,10^ A recent meta-analysis found that the use of this combination resulted in greater urine output, particularly in patients with lower baseline serum albumin levels.^10^ Conversely, bumetanide, which has a lower binding affinity to albumin,^4^ may be less affected by hypoalbuminemia.^11^ Bumetanide is less impacted because a larger proportion of the diuretic remains unbound and available for secretion into the proximal tubule to exert pharmacological activity.^4^ However, the pharmacokinetics of bumetanide remains debated in critically ill patients with hypoalbuminemia.

The 2024 CHEST Critical Care Consensus Statement on the Utilization of Intravenous Albumin strongly recommended against the use of albumin with diuretics, given lack of consistent benefit.^12^ The guidelines primarily evaluated literature that involved bolus loop diuretic administration in addition to albumin boluses rather than continuous infusions as a method of overcoming diuretic resistance.^1,9,13^ Notably, there is a lack of studies directly comparing the co-administration of albumin with furosemide versus bumetanide. This study aims to evaluate urine output response with continuous infusions of bumetanide compared with furosemide-albumin in critically ill patients.

## Methods

### Study Design

This was a single-center, retrospective cohort study conducted within a single hospital system comprising 1 academic medical center and 6 community hospitals. Patients were included if they were admitted to an ICU between January 1, 2022 and January 1, 2024, and received either a furosemide-albumin or bumetanide continuous infusion for at least 24 hours, with a Foley catheter in place prior to diuretic initiation. Exclusion criteria included nephrotic syndrome, defined as a random urine protein-to-creatinine ratio greater than 3000 mg/g or a past medical history of nephrotic syndrome based on ICD-10 codes or documentation, end-stage renal disease, or severe renal impairment, defined as a serum creatinine level greater than 4 mg/dL.^5,14^ Furosemide-albumin was compounded using furosemide 10 mg/mL vials to achieve a final furosemide concentration of 1 mg/mL using 50 mL of albumin 25% or 2 mg/mL using 100 mL of albumin 25%. An equivalent volume of albumin was withdrawn prior to adding furosemide to maintain the labeled albumin volume and concentration. Bumetanide was compounded using bumetanide 10 mg/mL vials and diluted in either 100 mL of dextrose 5% or normal saline to achieve final bumetanide concentrations of 0.05 or 0.1 mg/mL. Standardized dilution is required per our institution’s pharmacy policy. The study was approved by the local institutional review board.

### Outcomes

The primary endpoint was cumulative urine output during the first 24 hours following the initiation of the continuous diuretic infusion. Secondary endpoints at 24 hours after diuretic initiation included total loop diuretic dose (calculated in intravenous [IV] furosemide equivalents;^15,16^ 1 mg IV bumetanide equivalent to 20 mg IV furosemide), net fluid balance, total albumin bolus dose administered, serum creatinine, serum albumin levels, ICU length of stay, and in-hospital mortality. A cost estimate was conducted using the wholesale acquisition costs (WACs) at our institution. The WAC for a 100 mL bag of bumetanide 0.1 mg/mL was $16.52, and the WAC for a 100 mL bag of furosemide 2 mg/mL in albumin 25% was $119.54. The total cost was determined by multiplying the number of bags required in the first 24 hours, based on the total dose administered, by the cost per bag of the respective diuretic.

### Statistical Analysis

Categorical variables were compared using the chi-square test and presented as counts and percentages. Continuous variables were compared using the Mann-Whitney *U* test and presented as medians with interquartile ranges (IQRs). A multivariable linear regression model was used to analyze the primary outcome and to adjust for potential confounders. The covariates were assessed in an initial univariable analysis and included baseline serum creatinine and serum albumin levels, heart failure, and receipt of an albumin bolus, inotropes, or thiazide-like diuretics during diuretic infusion. Variables that introduced high multicollinearity, as evidenced by variance inflation factors (VIFs) exceeding a threshold of 5, were excluded from the final model. All analyses were performed using Minitab version 21 (Minitab LLC, State College, Pennsylvania). A 2-sided *P* value of less than 0.05 was considered statistically significant.

## Results

A total of 557 patients were evaluated for inclusion. Of these, 123 were excluded based on study criteria, 66 were in the bumetanide group and 56 were in the furosemide group, leading to 434 patients in the final analysis (Figure 1). A total of 173 patients received a continuous infusion of bumetanide, and 261 patients received a continuous infusion of furosemide-albumin. Baseline characteristics are presented in Table 1. The median age was 66 years, and more than half of the patients were male and White. A greater proportion of patients in the bumetanide group had a past medical history of chronic kidney disease (CKD) and heart failure. Laboratory parameters at the time of diuretic infusion initiation were significant for a lower creatinine clearance, as well as higher blood urea nitrogen (BUN) and serum albumin levels in the bumetanide group.

**Figure 1.**
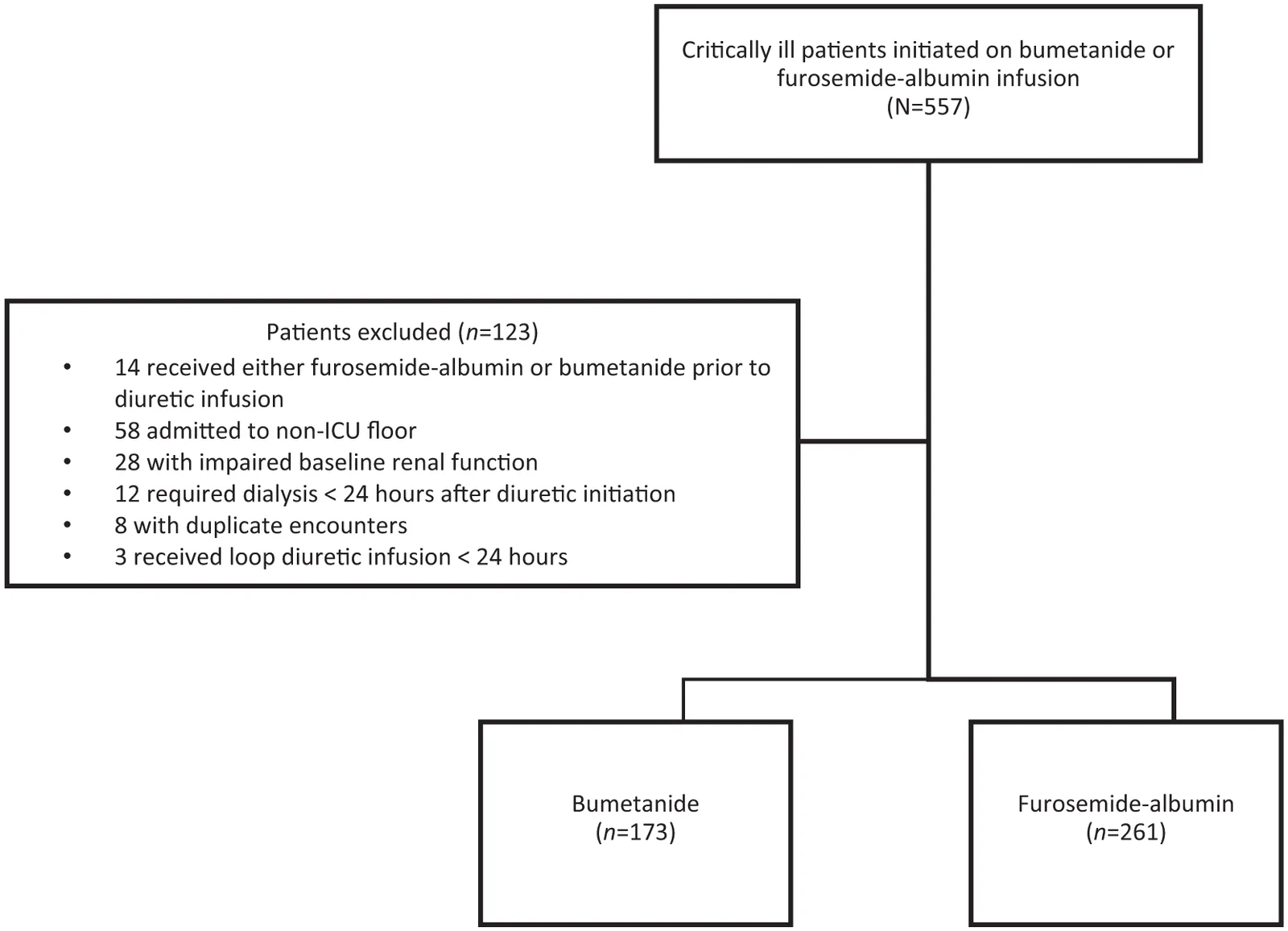
Patient inclusion.

**Table 1.** Patient Characteristics at Baseline.

Characteristic	Bumetanide (*n* = 173)	Furosemide-albumin (*n* = 261)	*P* value
Age (years), median (IQR)	66 (56-75)	67 (56-75)	0.866
Sex, *n* (%)			
Female	82 (47)	110 (42)	0.281
BMI (kg/m^2^), median (IQR)	30.1 (25.1-37.4)	29.9 (25.0-34.8)	0.507
Race, *n* (%)			
Asian	8 (5)	16 (6)	0.497
White	110 (63)	175 (67)	0.304
Black	50 (29)	53 (20)	0.014
Native American	0 (0)	1 (1)	0.519
Other^a^	5 (3)	16 (6)	0.092
Comorbidities, *n* (%)			
Chronic kidney disease	140 (81)	151 (58)	<0.001
Cirrhosis	23 (13)	35 (13)	0.972
Heart failure	152 (88)	176 (67)	<0.001
Prior to admission diuretics, *n* (%)			
Loop	53 (31)	51 (20)	0.008
Potassium-sparing	10 (6)	15 (6)	0.988
Thiazide	3 (2)	5 (2)	0.890
Laboratory parameters prior to diuretic initiation, median (IQR)			
Creatinine (mg/dL)	1.90 (1.2-2.42)	1.32 (0.3-1.74)	<0.001
Creatinine clearance (mL/min)	44.52 (27.26-57.64)	75.42 (41.34-92.69)	<0.001
eGFR (mL/min/1.73m^2^)	43 (25-53)	64 (39-86)	<0.001
BUN (mg/dL)	49 (31-62)	39 (25-50)	<0.001
Albumin (g/dL)	2.7 (2.3-3.1)	2.5 (0.9-2.9)	<0.001
Sodium (mEq/L)	136 (131-140)	139 (135-143)	<0.001
NT-proBNP (pg/mL)^b^	12426 (2346-14375)	8087 (1036-9520)	0.048

Abbreviations: BMI, body mass index; BUN, blood urea nitrogen; eGFR, estimated glomerular filtration rate; IQR, interquartile range; NT-proBNP, N-terminal pro-B-type natriuretic peptide.

a Other includes declined and unavailable.

b NT-proBNP values were available for 139 patients at baseline (67 in the bumetanide group and 72 in the furosemide-albumin group).

Primary and secondary outcomes are presented in Table 2. There was no significant difference in urine output between the 2 groups. However, the bumetanide group demonstrated a significantly greater net fluid loss, as reflected by a more negative fluid balance. Total diuretic dose (expressed in furosemide equivalents) was significantly higher in the bumetanide group. Change in serum creatinine at 24 hours after diuretic initiation was similar between the 2 groups. The furosemide-albumin group showed a significantly greater increase in serum albumin. Median ICU length of stay was significantly shorter in the bumetanide group. There was no difference in mortality rates at discharge.

**Table 2.** Primary and Secondary Outcomes^a^.

Outcome	Bumetanide (*n* = 173)	Furosemide-albumin (*n* = 261)	*P* value
**Primary outcome, median (IQR)**			
Urine output (mL)	3578 (1818-4738)	3617 (2035-4703)	0.304
**Secondary outcomes, median (IQR)**			
Total diuretic dose (mg)^b^	481 (295-544)	177 (109-236)	<0.001
Change in serum creatinine (mg/dL)	0.08 (–0.19 to 0.34)	0.10 (–0.07 to 0.21)	0.597
Net fluid balance (mL)	–1623 (–2921 to −177)	–1249 (–2365 to 146)	<0.001
Change in serum albumin (g/dL)	–0.07 (–0.3 to 0.2)	0.62 (0.2-0.9)	<0.001
ICU length of stay (days)	18 (5-23)	29 (10-34)	<0.001
Mortality rate at discharge, *n* (%)	32 (18)	58 (22)	0.346

Abbreviations: ICU, intensive care unit; IQR, interquartile range.

a Outcomes were evaluated from the time of the loop diuretic infusion initiation and until 24 hours after.

b Dose is presented in furosemide equivalents.

Laboratory parameters 24 hours after the initiation of the diuretic infusion are presented in Table 3. Creatinine clearance was significantly lower in the bumetanide group, with higher creatinine and BUN levels. Serum albumin was significantly higher in the furosemide-albumin group, consistent with the greater increase previously observed at 24 hours. Concomitant medications administered during the infusion are summarized in Table 4. Inotropes and thiazide/thiazide-like diuretics were more commonly administered in the bumetanide group, whereas loop diuretic boluses were more frequently used in the furosemide-albumin group. Supplemental albumin boluses, excluding albumin doses from the furosemide-albumin infusion, were more commonly administered in the bumetanide group compared with the furosemide-albumin group (*P* < 0.001).

**Table 3.** Laboratory Parameters 24 Hours After Initiation of Loop Diuretic Infusion.

Laboratory parameters, median (IQR)	Bumetanide (*n* = 173)	Furosemide-albumin (*n* = 261)	*P* value
Creatinine (mg/dL)	1.98 (1.31-2.44)	1.43 (0.80-1.88)	<0.001
Creatinine clearance (mL/min)	32 (18-42)	83 (44-96)	<0.001
eGFR (mL/min/1.73m^2^)	41 (25.0-52.0)	32 (56-82)	<0.001
BUN (mg/dL)	55 (36-68)	43 (28-57)	<0.001
Albumin (g/dL)	2.6 (2.2-3.0)	3.1 (2.6-3.5)	<0.001
Serum sodium (mEq/L)	137 (133-141)	141 (137-145)	<0.001
NT-proBNP (pg/mL)^a^	11 186 (2387-18 206)	7982 (2046-8194)	0.180

Abbreviations: BUN, blood urea nitrogen; eGFR, estimated glomerular filtration rate; IQR, interquartile range; NT-proBNP, N-terminal pro-B-type natriuretic peptide.

a NT-proBNP levels were available for 96 patients (33 in the bumetanide group and 63 in the furosemide-albumin group).

**Table 4.** Concomitant Medications During Diuretic Infusion.

Concomitant medications, *n* (%)	Bumetanide (*n* = 173)	Furosemide-albumin (*n* = 261)	*P* value
Albumin bolus	44 (25)	35 (13)	0.001
Total albumin dose (g)^a^, median (IQR)	10.9 (0-12.5)	43.7 (27.5-58.9)	<0.001
Antihypertensives	46 (27)	88 (34)	0.116
Inotropes	73 (42)	72 (28)	0.002
Loop diuretic bolus	2 (1)	37 (14)	<0.001
Potassium-sparing diuretic	14 (8)	17 (7)	0.532
Thiazide diuretic	58 (34)	54 (21)	0.003
Vasopressors	59 (34)	98 (38)	0.465

Abbreviation: IQR, interquartile range.

a Total albumin dose included albumin from furosemide-albumin as well as any additional supplementation.

After adjustment for potential confounders, the multivariable linear regression model revealed that baseline creatinine clearance and inotrope use were significantly associated with an increased urine output, whereas albumin bolus administration was associated with decreased urine output (Table 5). Neither bumetanide nor other variables, including heart failure, baseline serum albumin, and thiazide use/thiazide-like use, had a significant effect on urine output.

**Table 5.** Univariate and Multivariate Analyses.

	Univariate analysis		Multivariable analysis	
	Regression coefficient (β)	*P* value	Regression coefficient (β) (95% CI)	*P* value
History of heart failure	–455	0.059	–470 (–965, 24)	0.062
**Baseline laboratory parameters**				
Albumin^a^	–141	0.513		
Creatinine clearance (mL/min)	11.49	<0.001	12.29 (7.81, 16.76)	<0.001
**Medications while on diuretic infusion**				
Albumin bolus^b^	–1212	0.035	–1099 (–1885, –313)	0.006
Bumetanide	–39	0.853	356 (–79, 791)	0.108
Inotropes	461	0.036	697 (260, 1135)	0.002
Thiazide diuretics	198	0.403	356 (–79, 791)	0.108

a Baseline serum albumin >2.5 g/dL as comparator versus <2.5 g/dL as reference.

b Albumin bolus administration >0.5 mg/kg/day as comparator versus <0.5 mg/kg/day as reference.

Regarding cost, based on the 24-hour diuretic use and WAC, bumetanide 0.1 mg/mL in 100 mL bag cost $49.56 (IQR = $33.04-$49.56) whereas furosemide 1 mg/mL in albumin 25% 100 mL cost $119.54 (IQR = $119.54-$239.08).

## Discussion

Existing literature on continuous loop diuretic infusions for diuretic resistance has primarily focused on comparing furosemide-albumin infusions to furosemide alone, with mixed results.^9,10^ Enhanced diuresis has been mainly demonstrated in patients with nephrotic syndrome and those with a serum albumin level <2.5 g/dL.^10^ In contrast, bumetanide’s diuretic efficacy has not been shown to be significantly affected by hypoalbuminemia,^11^ which may make it a viable alternative for critically ill patients with diuretic resistance. This is particularly important as albumin administration carries concerns, such as anaphylaxis, infection risk, potential transient volume expansion in hypervolemic patients, and a high cost.^10^

To our knowledge, this is the first study to directly compare the clinical effects of continuous bumetanide versus furosemide-albumin infusions in critically ill patients. Our findings indicate that there was no significant difference in urine output at 24 hours between the 2 continuous diuretic infusion regimens. Interestingly, the bumetanide group required a 4-fold higher dose in comparison with the furosemide-albumin group to achieve comparable urine output. This finding aligns with a previous study by Ng et al,^17^ which reported a similar median furosemide infusion (without albumin) dose of 168 mg and bumetanide infusion dose of 480 mg to achieve a comparable urine output. The higher bumetanide dose in our study may be attributed to differences in baseline characteristics, including the higher incidence of CKD and heart failure, and lower creatinine clearance, all of which could predispose patients to an increased risk of diuretic resistance. Notably, we excluded patients with nephrotic syndrome, end-stage renal disease, and severe renal impairment to minimize confounding as these conditions have been shown to alter loop diuretic pharmacokinetics and diminish responsiveness.^5^ In addition to urine output, net fluid balance has also been used as an indicator for vascular decongestion, particularly in critically ill patients.^18,19^ In line with the findings of Ng et al,^17^ bumetanide was associated with a significantly more negative fluid balance when compared with furosemide-albumin.

Limited data focus specifically on the utilization of bumetanide in fluid accumulated ICU patients, and available literature evaluating the impact of albumin on ICU length of stays and mortality is conflicting.^1,13,20^ For example, similar to our findings, Côté et al^6^ observed a longer ICU lengths of stay with the use of albumin in critically ill patients receiving loop diuretics. However, unlike the aforementioned study, we did not observe a difference in mortality rates between the 2 groups. To explore factors influencing diuretic response, we conducted a multivariable regression analysis. After adjusting for potential confounders, lower baseline creatinine clearance was identified as an independent predictor of decreased urine output. Of note, the bumetanide group had worse baseline renal function, as evidenced by lower creatinine clearance, higher creatinine, and higher BUN levels. The higher prevalence of renal dysfunction in this group likely reflects confounding by indication, as bumetanide is often preferred in patients with baseline renal impairment or suspected diuretic resistance. This selection bias may have influenced comparisons of dose requirements and clinical response between the groups, and likely accounts for the higher diuretic doses observed in the bumetanide group. While heart failure, which was more common in the bumetanide group, is linked to diuretic resistance, it did not significantly affect urine output in our model. This is in line with a previous study of non-ICU patients with acute heart failure demonstrating similar urine output with continuous infusions of bumetanide and furosemide.^17^ Baseline albumin was also evaluated in the model but did not emerge as a predictor of urine output. We used an albumin cut-off of 2.5 g/dL, as literature suggests limited utility of albumin co-administration in patients with higher albumin levels.^10^ While hypoalbuminemia can theoretically impair the tubular secretion of protein-bound diuretics, its lack of significance in our multivariable model suggests that urine output was not primarily affected by albumin-mediated diuretic resistance. This may reflect the concurrent administration of albumin in the furosemide group to mitigate these pharmacokinetic limitations, as well as bumetanide’s maintained efficacy across the varying ranges of albumin levels.” Thiazide diuretic use did not significantly affect urine output in our study. Consistent with previous literature, inotropes were an independent predictor of increased urine output.^21^ Interestingly, the administration of an albumin bolus was associated with lower urine output. In this study, patients in the bumetanide group were more likely to receive albumin boluses, despite having a higher baseline albumin and bumetanide’s lower reliance on albumin for efficacy. However, we cannot confirm whether albumin boluses were administered for diuresis enhancement rather than treatment of hypovolemia or other complications within the critically ill patient population, which may explain the observed lower urine output. This study is not without limitations. Firstly, the retrospective nature of the study precludes the establishment of a causal relationship and limits our ability to elucidate all factors potentially influencing diuretic response. Furthermore, incomplete documentation, such as missed fluid intake or urine outputs may have introduced measurement bias, although we attempted to limit this by only including patients with a Foley catheter present during diuretic infusion. In addition, certain patient populations may not accept blood products including albumin and this was unaccounted for. Because the primary focus of the study was to evaluate diuretic efficacy, the study was not powered to assess related clinical markers; therefore, we were unable to assess whether this difference in fluid balance translated into improved clinical outcomes. Finally, there may be several unmeasured confounding variables, such as severity of illness or differences in diuretic titration strategies between providers, which influenced outcomes between the 2 groups.

## Conclusion and Relevance

Comparable urine output was seen in critically ill patients that received either continuous bumetanide infusion or continuous furosemide-albumin infusion particularly in patients with diuretic resistance and renal impairment. This study serves as a bridge to future trials to evaluate diuretic strategies in critically ill patients with CKD to determine the most effective approach in optimizing diuresis in the context of diuretic resistance, and to mitigate challenges presented by heterogeneous populations.
